# Self-cleaning liner for halogenated hydrocarbon control in landfill leachate

**DOI:** 10.1038/s41598-017-14562-y

**Published:** 2017-10-26

**Authors:** Shichong He, Lizhong Zhu

**Affiliations:** 1 Department of Environmental Science, Zhejiang University, Hangzhou, Zhejiang, 310058 China; 2 Zhejiang Provincial Key Laboratory of Organic Pollution Process and Control, Hangzhou, Zhejiang, 310058 China

## Abstract

Sorptive landfill liners can prevent the migration of the leachate pollutants. However, their sorption ability will decrease over time. A method should be developed to maintain the sorption ability of landfill liners. In this study, we combined cetyltrimethylammonium bromide-bentonite (CTMAB-bentonite) and zero-valent iron (ZVI) to develop a self-cleaning liner that can retain its sorption ability for a long period. Batch experiments and calculation simulations were employed to analyse the sorption ability of this liner material and the ecological risk of halogenated hydrocarbons. The results showed that CTMAB-bentonite could sorb halogenated hydrocarbons well, with saturated sorption capacities (*Q*_m_) of 10.2, 14.5, 6.69, 18.5, 29.4, and 49.7 mg·g^-1^ for dichloroethane (DCA), trichloroethane (TCA), dichloroethene (DCE), trichloroethylene (TCE), tetrachloroethylene (PCE), and 1,3- dichloropropene (1,3-DCP), respectively. Using the mixture of 0.5 g iron and 0.5 g CTMAB-bentonite could dramatically increase the removal efficiency of DCE, TCE, and PCE. The reaction with ZVI did not change the structure of CTMAB-bentonite and its sorption ability remained consistent. Calculation results suggested that the self-cleaning landfill liner would dramatically decrease the hazard index (HI) of the eluate. However, the humic acid and salt in leachate would cause a reduction in the removal of halogenated hydrocarbons.

## Introduction

Landfill leachate, containing various pollutants^1^, is the second largest pollution source in a landfill. Although the currently used liners can control hydraulic movement, the pollutants in leachate can also diffuse through the liner. Halogenated hydrocarbons are one of the most toxic groups of pollutants in leachate^2^ and they persist for an extremely long period in groundwater^3^. Groundwater is one of the main water sources in some regions. The ingestion of halogenated hydrocarbon affects several organs, and the liver is the most commonly affected organ. To prevent the migration of pollutants in leachate to groundwater, a sorptive landfill liner was developed in the present study^4^.

The purpose of a sorptive liner is to incorporate strong sorbents into a landfill liner^4^. The applied sorbents include active carbon^5,6^, natural zeolite^7^, organically modified kaolin^8^, and hexadecyltrimethyl ammonium bromide (HDTMA)-bentonite^9^. Among these sorptive materials, organobentonite was reported to be an excellent sorbent to sorb various organic pollutants from the aqueous phase^10–15^. The sorption of organic chemicals to the interlayer of organobentonite synthesized using long alkyl-chain surfactants (for example, cetyltrimethylammonium bromide, CTMAB) was recognized to be mainly via partitioning^10,14^. However, after a long period of landfill operation, the sorption ability of organobentonite can decrease gradually. Thus, it is necessary to find a way to remove the sorbed halogenated hydrocarbons and renew the sorption ability of CTMAB-bentonite.

Since Gillham and O’Hannesin utilized zero-valent iron (ZVI) for the remediation of groundwater^16^, the application of ZVI in the dehalogenation of pollutants has received considerable attention^17–22^. Iron has a redox potential of −0.44 V^23^, which is much lower than that of most halogenated hydrocarbons^24^, making it an effective reductant under anoxic conditions. ZVI has been employed to remediate carbon tetrachloride, dichloromethane, chloroform, and dichloroethene^25–27^. Moreover, the corrosion products of ZVI during the reduction, mainly Fe^2+^, are a more favourable reductive agent for halogenated hydrocarbons since they have a lower electrode potential of Fe_(s)_^3+^/Fe_(s)_^2+^, which is between −0.35 V and −0.65 V^23^. The addition of ZVI to the landfill liner would allow the sorbed halogenated hydrocarbons to be degraded. Thus, the sorption ability of organobentonite in the landfill liner could be recovered.

Therefore, this study aimed to develop a self-cleaning landfill liner to control the risk of halogenated hydrocarbons in leachate. We chose six typical halogenated hydrocarbons and investigated their sorption behaviour to CTMAB-bentonite. We also examined the degradation of three halogenated hydrocarbons by the addition of ZVI. The concentration change was simulated and the hazard index (HI) was calculated as well. The results of this work could provide essential data for building a self-cleaning landfill liner.

## Results and Discussion

### Sorption characteristics of halogenated hydrocarbons to CTMAB-bentonite

CTMAB-bentonite was shown to be an excellent sorbent in removing halogenated hydrocarbons, with *Q*_m_ values of 10.2, 14.5, 6.69, 18.5, 29.4, and 49.7 mg·g^−1^ for dichloroethane (DCA), trichloroethane (TCA), dichloroethene (DCE), trichloroethylene (TCE), tetrachloroethylene (PCE), and 1,3-dichloropropene (1,3-DCP), respectively. These results indicated that CTMAB-bentonite would be a suitable material for use in functional landfill liners to prevent the leakage of pollutants from the leachate to the groundwater. The sorption of halogenated hydrocarbons to CTMAB-bentonite was affected by their chemical structures. Chemicals with higher chlorine substitution values maintained larger *K*_L_ values, mainly because the long alkyl chain of CTMAB affected the sorption ability of these chemicals. Chemicals with higher chlorine substitution are more hydrophobic, resulting in a larger lg*K*_ow_ value (Table 1) and enabling them to be easily sorbed to CTMAB-bentonite.

**Table 1:** Octanol-water partition coefficient and the sorption coefficient of six halogenated hydrocarbons to CTMAB-bentonite.

Chemicals	lg*K*_ow_	*K*_L_ (L·kg^−1^)	*Q*_m_ (mg·g^−1^)	*K*_d_ (L·kg^−1^)
DCA	1.79	4.621	10.2	40.70
TCA	2.01	10.16	14.5	115.3
DCE	2.13	3.757	6.69	22.18
TCE	2.42	4.873	18.5	82.65
PCE	2.97	10.11	29.4	326.3
1,3-DCP	2.29	2.952	49.7	137.3

The sorption of halogenated hydrocarbons to CTMAB-bentonite is limited by the unsaturated bonds. For example, when DCA is compared with DCE, in which a single bond is replaced by a double bond, the *K*_L_ value is decreased from 4.62 to 3.75. A similar trend was found for TCA and TCE, in which the *K*_L_ value of the two halogenated hydrocarbons decreased from 10.6 to 4.87, respectively. These differences could be attributed to the different structure of chemicals used in this experiment, which would affect the sorption force of them to organobentonite^28^. For aliphatic compounds, the interaction mechanism is mainly dominated by the cavity/dispersion interaction^29^. Compared DCA and DCE, TCA and TCE, when their saturated bond was placed with an unsaturated bond, the cavity/dispersion interaction between DEC or TCE with CTMAB cations became much larger (Table S2), resulted in a higher removal rate. As a result, although halogenated hydrocarbons with unsaturated bonds have a higher lg*K*_ow_ values, their *K*_L_ values when sorbed to CTMAB-bentonite were lower.

### Degradation of halogenated hydrocarbons and self-cleaning of landfill liner

**Figure 1:**
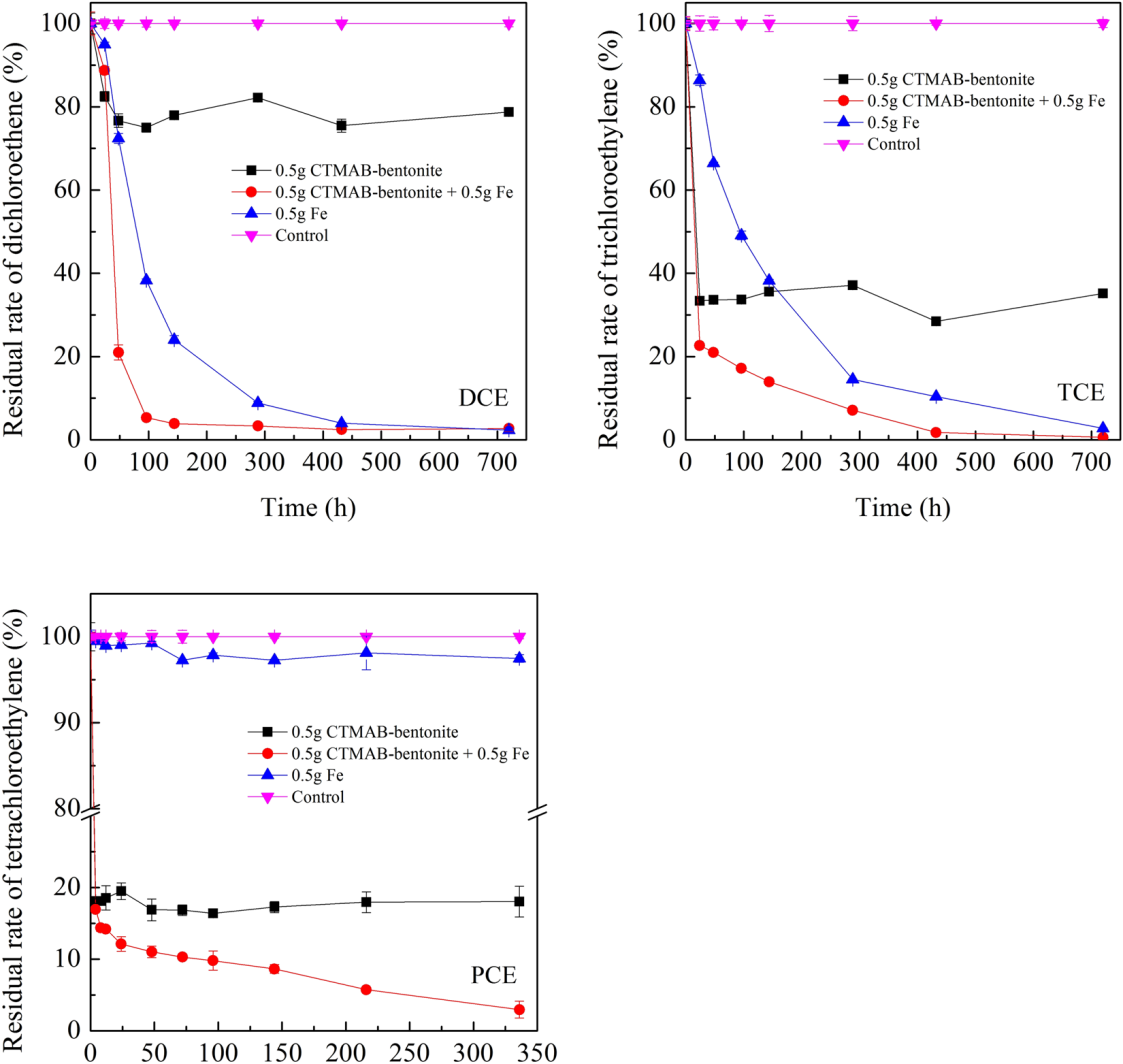
Removal of DCE, TCE, and PCE by CTMAB-bentonite, iron and the mixture of CTMAB-bentonite and iron.

To recover the sorption ability of CTMAB-bentonite and achieve self-cleaning of the landfill liner, we utilized ZVI to remove the sorbed halogenated hydrocarbons from CTMAB-bentonite. As displayed in Fig. 1, the concentrations of DCE, TCE, and PCE decreased rapidly in the first few hours and then reached an equilibrium concentration. DCE was removed rapidly by CTMAB-bentonite, with a removal efficiency of approximately 20% in one hour. DCE removed by iron powder was much slower, and the residual amount was approximately 2.5% after a reaction time of longer than 400 hours. The residual concentration of DCE treated by the mixture of 0.5 g CTMAB-bentonite and 0.5 g iron powder first dropped rapidly in the first few hours and then slowed when the reaction period was longer than 50 hours. When the reaction period was longer than 100 hours, DCE reached the equilibrium state and its residual amount was approximately 2.4–3.3%. The removal of TCE showed a similar trend. The removal rate of TCE could exceed 65% when it was sorbed to CTMAB-bentonite, while the residual amount was extremely low when it reacted with iron powder (2.75%) or the mixture of CTMAB-bentonite and iron powder (0.62%). The reactions of iron with DCE or TCE were completed very quickly. As a result, the removal of DCE and TCE was mainly due to their reaction with iron, and no synergistic effects were observed. PCE could be removed efficiently by CTMAB-bentonite at a removal rate of more than 80%. However, its reaction with iron was negligible and only approximately 2% of PCE was removed. A synergistic reaction occurred when CTMAB-bentonite and iron mixed together. The removal rate of PCE by the mixture was higher than 97% when the reaction time was longer than 330 hours.

The removal of halogenated hydrocarbons by CTMAB-bentonite and iron can be described according to the following two steps: (1) halogenated hydrocarbons were sorbed to CTMAB-bentonite; (2) the sorbed halogenated hydrocarbons reacted with iron when degraded, or iron reacted with halogenated hydrocarbons in the aqueous phase, and then, the sorbed chemicals in CTMAB-bentonite were further released to the solution. The sorption process was a chemical balance of the halogenated hydrocarbon distributed between the aqueous phase and the organic phase of CTMAB-bentonite. When one type of halogenated hydrocarbons was sorbed to CTMAB-bentonite, it became separated in the organic phase and then extended the layer space, resulting in an increase of the interlayer spacing. When the sorption reached the equilibrium condition, the concentration of the halogenated hydrocarbon in the aqueous phase or the organic phase became stable accordingly. If there was a persistent input source of halogenated hydrocarbon, the balance was disrupted and the chemicals tended to transfer into the organic phase in the interlayer of CTMAB-bentonite. With chemicals sorbed to CTMAB-bentonite continually, more energy will be required for the subsequent pollutants to enter the interlayer space. As a result, the sorption ability of CTMAB-bentonite will be weakened and finally restricted. With the addition of iron, the sorbed halogenated hydrocarbons can be degraded accordingly, the interlayer spacing of CTMAB-bentonite will decrease, and the sorption ability of CTMAB-bentonite can be partly recovered. Additionally, the sorbed halogenated hydrocarbons CTMAB-bentonite could provide a high initial concentration for the degradation procedure, providing a faster reaction speed.

**Figure 2:**
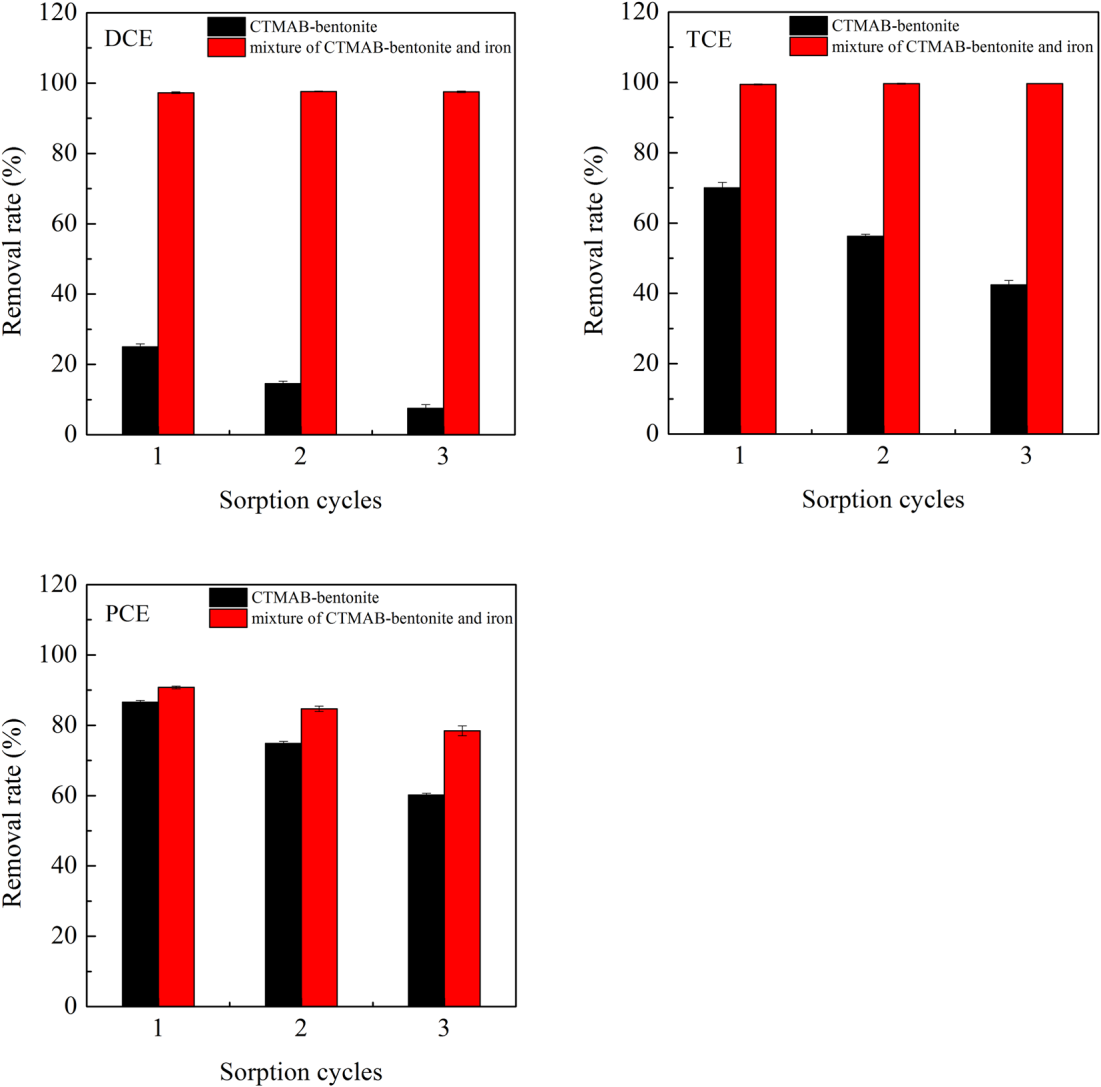
Repeat removal of DCE, TCE, and PCE by CTMAB-bentonite or the mixture of CTMAB-bentonite and iron.

As a self-cleaning landfill liner, its sorption ability should not decrease substantially over a long period. A repeated sorption procedure was applied to investigate the self-cleaning ability of the landfill liner material (Fig. 2). After three cycles of sorption, the percentage removal rate of three halogenated hydrocarbons sorbed to CTMAB-bentonite dropped from 24.99% to 7.51%, 70.02% to 42.37%, and 86.59% to 60.18% for DCE, TCE, and PCE, respectively. Correspondingly, the percentages of the three halogenated hydrocarbons removed by CTMAB-bentonite and iron mixture showed a slight change. The observed removal rates remained above 97% and 99% for DCE and TCE, respectively. Though the removal rate of PCE dropped, it showed a relatively lower degree of reduction (from 90.76% to 78.45%). These findings indicate that the halogenated hydrocarbons could be self-cleaned by this landfill liner material. Landfilling is a long-term process, and a liner with self-cleaning ability would better prevent the migration of halogenated hydrocarbons.

**Figure 3:**
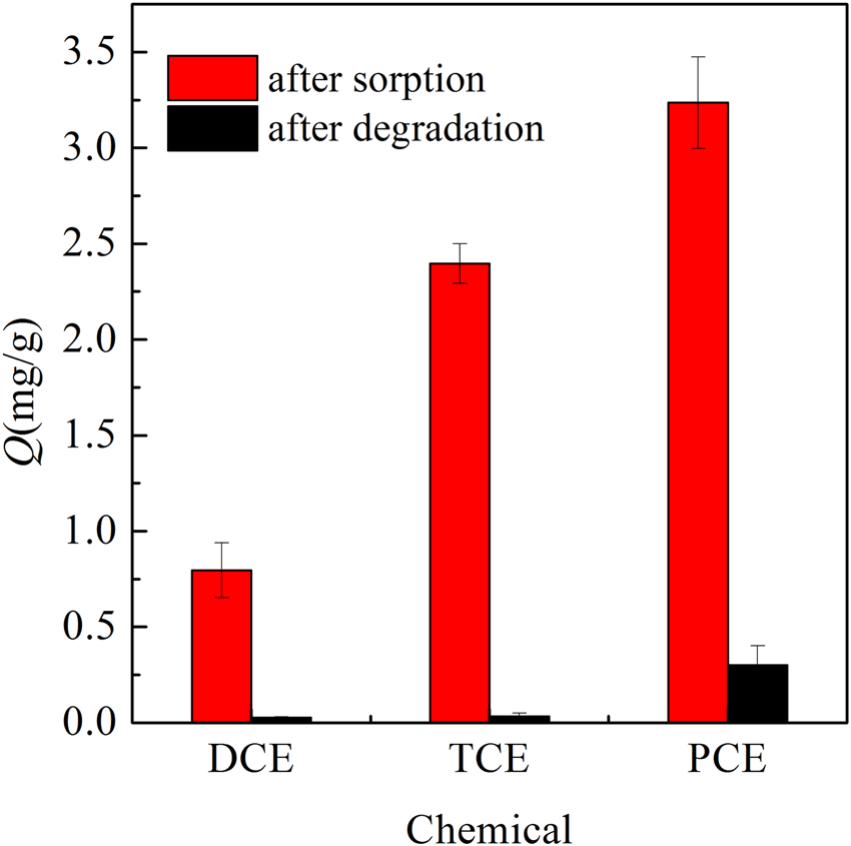
Halogenated hydrocarbons in CTMAB-bentonite after sorption or degradation.

To verify that the sorbed halogenated hydrocarbons were degraded, a series of 3-day TCE sorption samples were selected, and their amount was measured via sorbent extraction. As displayed in Fig. 3, the concentrations of DCE, TCE, and PCE were 0.80, 2.40, and 3.24 mg·g^−1^, respectively, in CTMAB-bentonite after the sorption procedure. These values corresponded to the amount calculated through the sorption isotherm. In contrast, the concentrations of DCE, TCE, and PCE dropped dramatically to 0.026, 0.033, and 0.300 mg·g^−1^, respectively, in CTMAB-bentonite when iron was present simultaneously (P < 0.05). This result indicated that the sorbed halogenated hydrocarbons were almost degraded during the experimental period. Note that the residual amount of PCE was not as low as that of DCE or TCE, which could be ascribed to its much smaller degradation rate. It could be deduced that PCE could be degraded completely if the experimental period was extended.

Additionally, X-ray diffraction (XRD) and Fourier transform infrared (FTIR) spectroscopy were conducted to evaluate the structural properties of CTMAB-bentonite after the degradation procedure. The XRD results could reveal the interlayer spacing^30^. The same peak position indicated that the interlayer spacing did not change for CTMAB-bentonite after degradation (Figure S1). According to previous reports, the *v*_as_ mode of the CH_2_ infrared absorption bands on FTIR patterns was sensitive to the concentration of the interlayer surfactant of CTMAB-bentonite^31–33^. When bentonite was synthesized using CTMAB, two peaks appeared at the wavelengths of 2850.3 and 2918.8 cm^−1^ (Figure S2). After sorption of TCE, these two peaks of CTMAB-bentonite shifted to a lower frequency of 2848.3 and 2916.7 cm^−1^. After degradation, these two peaks shifted back to the same frequency of 2850.3 and 2918.8 cm^−1^ for CTMAB-bentonite. It could be inferred that the amount of CTMA^+^ was the same for CTMAB-bentonite before and after the degradation procedure. Total organic content (TOC) values can be used to analyze the amount of organic carbon in CTMAB-bentonite. After sorption of halogenated hydrocarbons, the TOC value would increase accordingly. The similar TOC values of the two CTMAB-bentonites indicated that the sorbed halogenated hydrocarbons were degraded (Table S1). According to these results, we can conclude that the structure of CTMAB-bentonite remained the same and that its sorption ability did not decrease.

### Calculation of the degradation of halogenated hydrocarbons

As described in Table 2, the first-order kinetic coefficients (*k*) of DCE, TCE, and PCE reacted with 0.5 g iron powder were 0.0076, 0.0049, and 0.0003, respectively. This value increased to 0.0394, 0.0055, and 0.0046 for DCE, TCE, and PCE reacted with the mixture of 0.5 g iron and 0.5 g CTMAB-bentonite, respectively. The removal of halogenated hydrocarbons could be separated to be removed by CTMAB-bentonite and degraded by iron. As a result, the larger value of *k* can be primarily attributed to the sorption ability of CTMAB-bentonite, and the former values were adopted in the calculation. The calculated values fit well with the experimental results, indicating that this method can be used to determine the variation of halogenated hydrocarbon concentrations (Figure S3).

**Table 2:** The first-order kinetic coefficients (*k*) of DCE, TCE, and PCE removed by different materials.

Chemicals	*k* (h^−1^)	
	the mixture of CTMAB-bentonite and iron	0.5 g Fe
PCE	0.0046	0.0003
TCE	0.0055	0.0049
DCE	0.0394	0.0076

**Figure 4:**
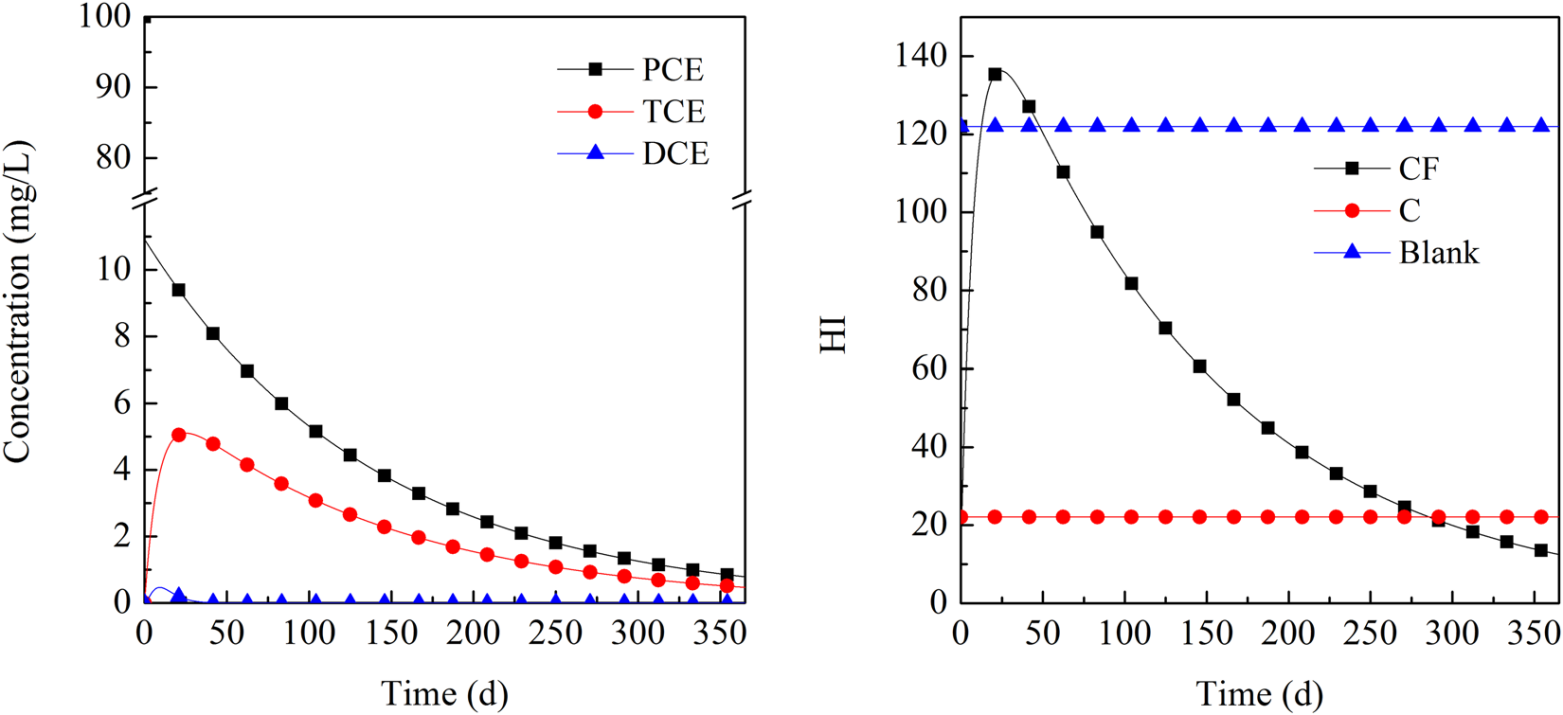
Calculated concentration changes of DCE, TCE, and PCE with an initial PCE concentration of 100 mg·L^−1^. (CF in the legend presents the HI value of solution treated using the mixture of CTMAB-bentonite and iron, C presents the HI value of solution treated using CTMAB-bentonite)

The values of DCE, TCE, and PCE are shown in Fig. 4 when the initial concentration of PCE was 100 mg·L^−1^. For the removal of PCE using the mixture of 0.5 g iron and 0.5 g CTMAB-bentonite, the concentration of PCE decreased rapidly in the first period. Then, the concentration dropped smoothly and continually. As the degradation products, the concentrations of TCE and DCE first showed an increase and then dropped. The concentration peak appeared on approximately the 25th day at 5.1 mg·L^−1^ and on approximately the 9th day at 0.47 mg·L^−1^ for TCE and DCE, respectively. Furthermore, their concentrations continually dropped as the reaction proceeded. The hazard index (HI) value of the blank set and the tubes with 0.5 g CTMAB-bentonite remained constant at 122 and 22.1, respectively. However, the HI of tubes with the mixture of 0.5 g iron and 0.5 g CTMAB-bentonite rose rapidly at first and reached the peak point of 136.2 on the 25th day, after which they also dropped rapidly. The HI further decreased and became lower than that in the blank set and tubes with 0.5 g CTMAB-bentonite on the 48th and the 286th day, respectively.

**Figure 5:**
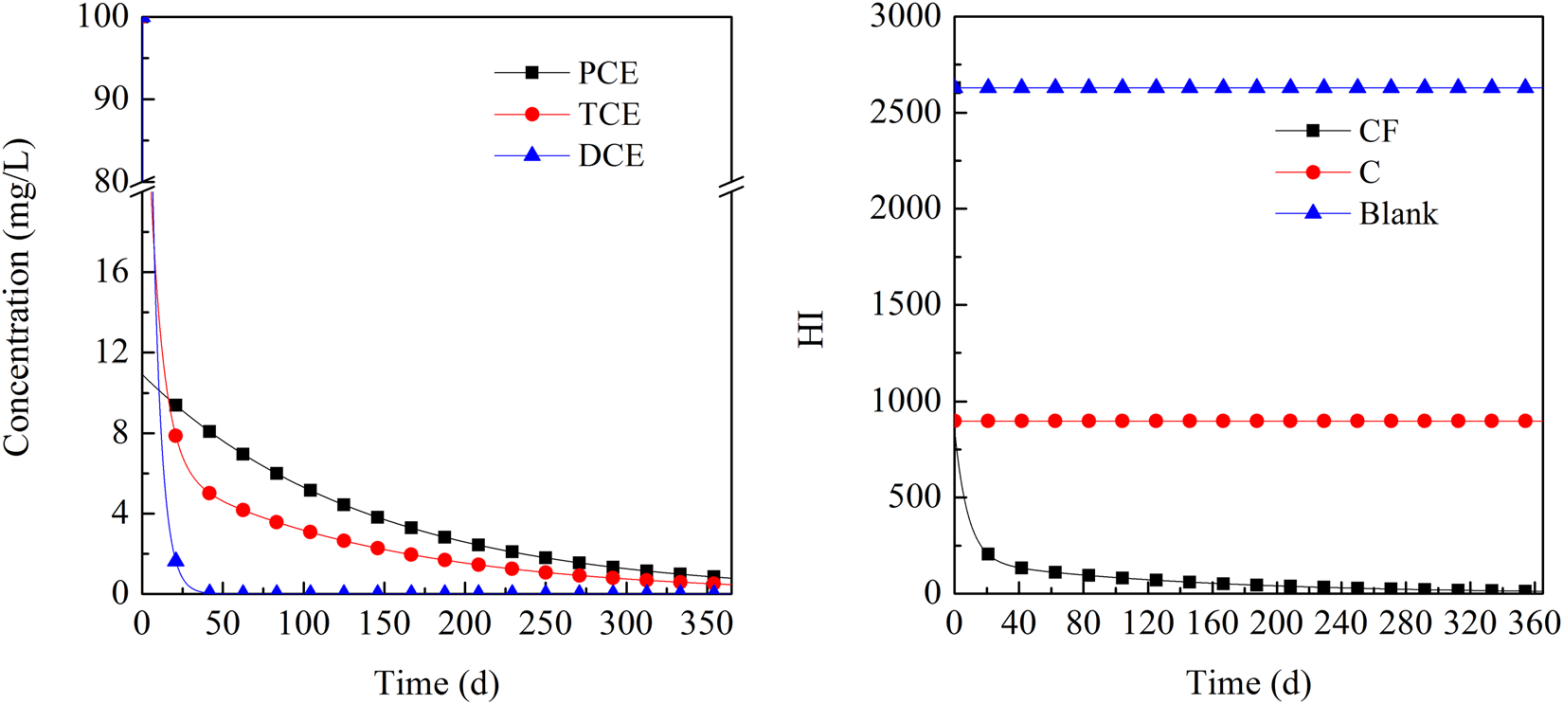
Calculated concentration changes of DCE, TCE, and PCE with an initial DCE, TCE, and PCE concentration of 100 mg·L^−1^ each. (CF in the legend presents the HI value of solution treated using the mixture of CTMAB-bentonite and iron, C presents the HI value of solution treated using CTMAB-bentonite)

When the concentrations of DCE, TCE and PCE were all 100 mg·L^−1^, the degradation of the three halogenated hydrocarbons appeared with different tendencies (Fig. 5). For tubes containing 0.5 g CTMAB-bentonite, the concentrations of the three halogenated hydrocarbons all dropped rapidly, and the rate was DCE > TCE > PCE. The HI of the blank set was 2630; it dropped to 898 in the presence of 0.5 g CTMAB-bentonite. The HI of tubes with the mixture of 0.5 g iron and 0.5 g CTMAB-bentonite dropped very quickly in the first period, followed by a subsequent gradual decrease, dropping to only 10% of the blank set by 15 days.

When PCE was present alone, the HI was not high; it was even lower in the treatment with only CTMAB-bentonite. When PCE began to degrade, a rapid increase in the HI was observed, which was attributed to the high toxicity of the degradation intermediate TCE. TCE has a large RfD value and is approximately two orders magnitude larger than PCE or DEC. As a result, even though its concentration was very low, it presented a high carcinogenic potential. The reaction of iron with DCE, TCE and PCE was correlated with their concentrations and reaction coefficients. In the first stage, PCE began to be degraded to TCE. The concentration of PCE was much larger than that of TCE. Moreover, the reaction coefficient of TCE was similar to that of PCE, which resulted in an increased concentration of TCE and a continued increase of the HI. As the reaction further proceeded, TCE with the larger reaction coefficient would be degraded somewhat faster, resulting in a further decrease of its concentration at time points after 25 days. As a result, the HI of the solution decreased accordingly.

If DCE, TCE and PCE were present simultaneously at the initial concentration of 100 mg·L^−1^, the HI would be extremely high at 2630. However, the CTMAB-bentonite would reduce the HI to one-third at 898. This response could be attributed to the non-competitive sorption of the three halogenated hydrocarbons to CTMAB-bentonite. All three halogenated hydrocarbons can be degraded by iron. As the reaction proceeded, the HI of the solution decreased accordingly.

The purpose of using a landfill liner is to prevent the leakage of leachate and prevent its further contamination to groundwater and the resulting hazardous consequences to people. If the solution could be kept in the reacting liner long enough, the halogenated hydrocarbons would be all degraded and no hazardous effect would be caused by the eluted solution. Furthermore, a thinner self-cleaning landfill liner would have a simulating effect on protecting groundwater from being polluted by the leached chemicals.

### Influence of humic acid and ion strength

**Figure 6:**
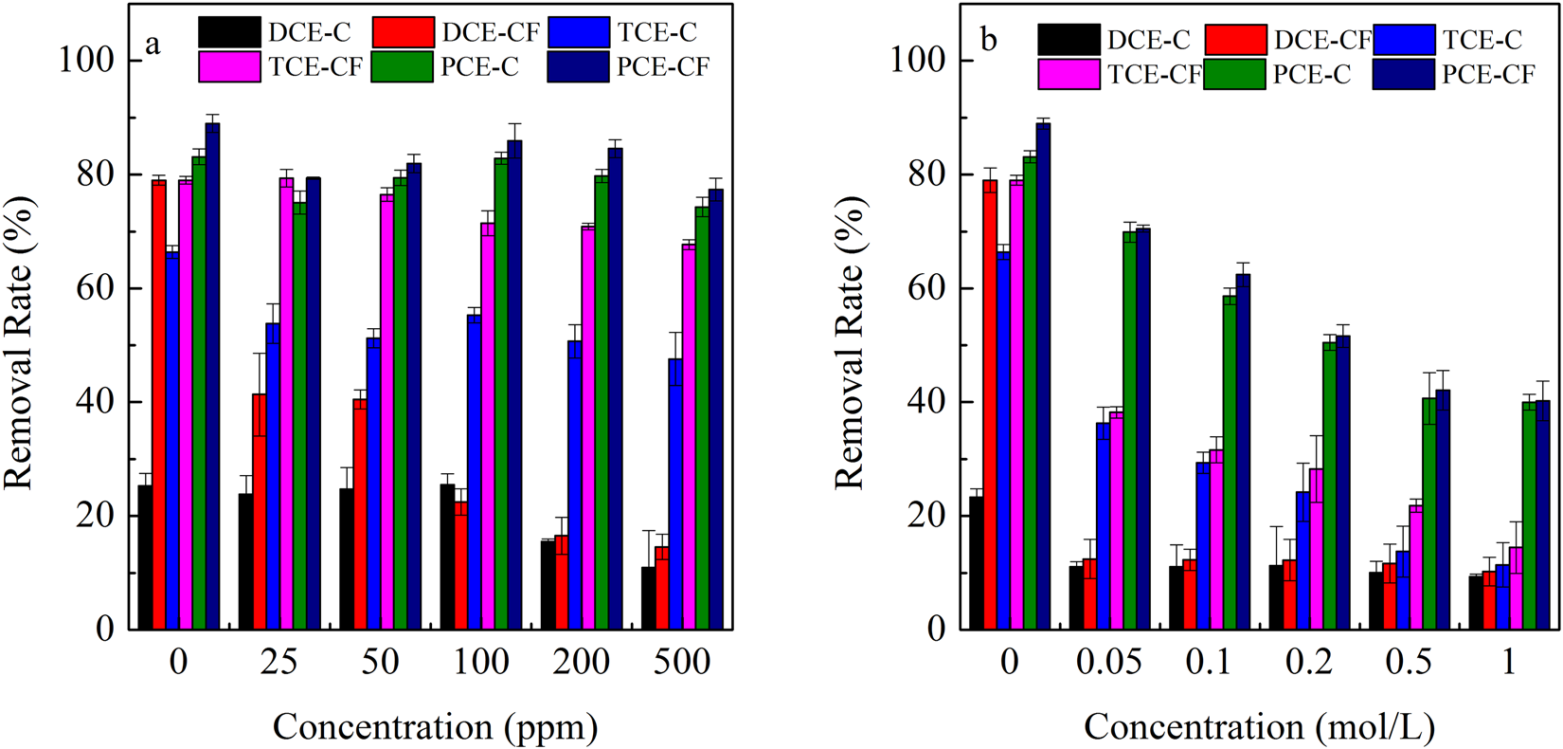
Effect of humic acid and ion strength on the removal rate of DCE, TCE, and PCE by CTMAB-bentonite or the CTMAB-bentonite-iron mixture. (**a**) presents the influence of humic acid; (**b**) presents the influence of ionic strength; the C in legend present the removal rate of chemicals by CTMAB-bentonite and CF present the removal rate of chemicals by the mixture of CTMAB-bentonite and iron)

The humic acid in the aqueous solution affected the sorption ability of halogenated hydrocarbons to CTMAB-bentonite as well as their degradation by ZVI. As the concentration of humic acid increased from 0 to 500 mg·L^−1^, the removal rate of DCE, TCE, and PCE sorbed to CTMAB-bentonite dropped from 25.3% to 10.9%, 66.4% to 47.6%, and 83.1% to 74.3%, respectively (Fig. 6). The removal rate dropped from 79.1% to 14.6%, 79.1% to 67.7%, and 88.9% to 77.4% for DCE, TCE, and PCE, respectively, with the CTMAB-bentonite and iron mixture.

Humic acid could be removed by CTMAB-bentonite efficiently in an aqueous phase. The sorbed humic acid could increase the sorption ability of halogenated hydrocarbons to CTMAB-bentonite. However, the residual amount of humic acid will increase the solubility of chemicals in the aqueous phase and result in a negative effect in the removal of halogenated hydrocarbons. This effect would counteract the increased ability of halogenated hydrocarbons to adsorb to CTMAB-bentonite. Furthermore, the humic acid in the aqueous phase might form an inner-sphere complex and thus inhibit the removal of pollutants by ZVI^19,34^. As a result, the removal rate of halogenated hydrocarbons decreased with the increase of humic acid in the aqueous phase. This process will further decrease the initial concentration of chemicals and have a negative effect on the degradation of halogenated hydrocarbons by iron. Moreover, the dissolved humic acid would occupy the reaction surface of iron, leading to a reduction in degradation efficiency.

Greater ionic strength decreased the removal rate of halogenated hydrocarbons by CTMAB-bentonite and the CTMAB-bentonite-iron mixture (Fig. 6). As the concentration of NaNO_3_ increased from 0 to 1 mol·L^−1^, the removal rate of DCE, TCE, and PCE sorbed to CTMAB-bentonite dropped from 25.1% to 9.37%, 66.2% to 11.4%, and 83.2% to 40.0%, respectively. The removal rate dropped from 79.0% to 10.2%, 79.0% to 14.5%, and 89.0% to 40.2% for DCE, TCE, and PCE, respectively, with the CTMAB-bentonite and iron mixture.

A high concentration of salt in aqueous solution confines the expansion of CTMAB-bentonite. Furthermore, the salt occupies the sorption site of the CTMAB-bentonite surface and the reaction site of iron surface, thus decreasing the sorption and degradation abilities.

## Materials and Methods

### Materials

The bentonite used in this research was obtained from Inner Mongolia, China. Its cation exchange capacity (CEC) was 1.084 mmol·g^−1^. The bentonite was ground to a powder smaller than 100 mesh and dried at 70 °C for 24 hours. CTMAB, ZVI powder, and six halogenated hydrocarbons, including DCA, TCA, DCE, TCE, PCE, and 1,3-DCP, were of analytical grade and were purchased from Aladdin Co. Ltd (Shanghai, China).

### Synthesis of organobentonite

A calculated amount of CTMAB, equal to 100% of bentonite’s CEC, was added to a glass beaker containing 400 mL deionized water and stirred at 70 °C until the CTMAB was dissolved. The solvent was then mixed with 20 g bentonite and stirred for 2 hours at 70 °C. The mixture was aged at 70 °C for 12 hours. The product was suction filtered and washed with deionized water several times until there was no precipitate in the filtrate when AgNO_3_ solvent was added. The final product was dried at 70 °C and then pulverized to pass through a 100-mesh sieve; it was denoted as CTMAB-bentonite.

### Sorption of halogenated hydrocarbon to CTMAB-bentonite

Each batch experiment was carried out using the standard batch equilibration technique performed according to an OCED guideline^35^. The details of sorption procedure and the operation of repeated sorption as well as the calculation of molecular interaction mechanisms are provided in the supplementary data. The influences of ion strength on the removal of halogenated hydrocarbons were studied by using a solution with an initial concentration of 0–100 mg·L^−1^ at 298 K; Na^+^ and NO_3_^−^ (0–1 mol·L^−1^) were used as representative ions. The influence of humic acid on the removal of halogenated hydrocarbons was tested at the solution concentration of 0–500 mg·L^−1^.

The Langmuir model was employed to delineate the experimental sorption isotherm to estimate the maximum capacity (*Q*_m_) of materials in sorption, and a linear model was used to obtain the sorption capacity (*K*_d_). The model descriptions are listed in the supplementary material.

### Degradation of halogenated hydrocarbon

Three separate batches were set to evaluate the effect of halogenated hydrocarbon degradation. Accordingly, 0.5 g of CTMAB-bentonite, the mixture of 0.5 g iron and 0.5 g CTMAB-bentonite, and 0.5 g of iron powder were placed in each tube of three separate batches. Then, after the addition of 20 mL of solution containing 100 mg·L^−1^ halogenated hydrocarbon, all the tubes were sealed with a Teflon cap and shaken at 25 °C, 150 rpm. One tube of each batch was selected at certain times, and the residual amount of halogenated hydrocarbon was analysed following the steps described in the sorption section. Solid samples were freeze-dried and followed ultrasonic extraction with hexane. The extraction efficiency for the tested compounds were 85% to 93%. For each batch, three duplications were set. Three control batches were set as well to evaluate the loss of chemicals via volatilization or adsorbed by the glass tube and Teflon cap.

### Calculation method for the concentration change and HI of halogenated hydrocarbons

The removal of halogenated hydrocarbons by the mixture of CTMAB-bentonite and iron was the sum of the halogenated hydrocarbons sorbed by CTMAB-bentonite and degraded by iron. The removal of halogenated hydrocarbons by different types of reagents can be described using the first-order kinetic model^36^, which is written as

1 $$C={C}_{0}\cdot {e}^{-kt}$$

where *C* represents the concentration of the chemical at time *t*, e is the natural logarithm, *k* (h^−1^) is apparent reaction coefficient, and *t* is time.

The degradation of halogenated hydrocarbons is based the consecutive reaction, and the concentration change of halogenated hydrocarbons as removed by CTMAB-bentonite and iron mixture could be calculated as follows. The details of the calculation are provided in the supplementary data.

2 $${C}_{PCE}={C}_{PCE,0}\cdot {e}^{-{k}_{1}\cdot t}\cdot \frac{V}{V+{K}_{d,PCE}\cdot m}$$

3 $${C}_{TCE}=[\frac{{C}_{PCE,0}\cdot {k}_{1}}{{k}_{2}-{k}_{1}}\cdot ({e}^{-{k}_{1}\cdot t}-{e}^{-{k}_{2}\cdot t})+{C}_{TCE,0}\cdot {e}^{-{k}_{2}\cdot t}]\cdot \frac{V}{V+{K}_{d,TCE}\cdot m}$$

4 $${C}_{DCE}=\{\frac{{C}_{PCE,0}\cdot [1-({k}_{2}\cdot {e}^{-{k}_{1}\cdot t}-{k}_{1}\cdot {e}^{-{k}_{2}\cdot t})]}{{k}_{2}-{k}_{1}}\cdot {e}^{-{k}_{3}\cdot t}+{C}_{DCE,0}\cdot {e}^{-{k}_{3}\cdot t}\}\cdot \frac{V}{V+{K}_{d,TCE}\cdot m}$$

HI can be used to estimate the possible toxic effects on humans^37^ and can be calculated as follows:

5 $${\rm{HI}}={\rm{CDI}}/{\rm{RfD}}$$

where RfD (the reference dose for the selected compounds, mg·kg^−1^·d^−1^) is a numerical estimate of a daily oral exposure to the human population, including sensitive subgroups such as children, that is unlikely to cause harmful effects during a lifetime^38^. CDI (chronic daily intake, mg·kg^−1^·d^−1^) can be calculated as^37^

6 $${\rm{CDI}}=[({C}_{{\rm{water}}}\times {\rm{WI}}\times {\rm{ED}}\times {\rm{EF}})/({\rm{BW}}\times {\rm{AT}})]$$

where *C*_water_ is the pollutant’s concentration in water, water intake (WI) = 2 L·day^−1^, exposure duration (ED) = 30 years, exposure frequency (EF) = 350 days·year^−1^, body weight of the target (BW) = 70 kg (adult), and exposure average time (AT) is 30 years for non-carcinogenic compounds or 70 years (lifetime) for carcinogenic compounds.

### Analytical methods

The halogenated hydrocarbon concentrations were measured using an Agilent gas chromatograph (Agilent 7890B, Agilent Technologies Inc., USA) equipped with an ECD detector and a capillary column (DB-5 MS). The limits of detection (LOD) of halogenated hydrocarbons were 1.61, 0.56, 7.19, 0.05, 0.02, and 0.65 μg·L^−1^ for DCA, TCA, DCE, TCE, PCE, and 1,3-DCP, respectively. The organic carbon content (*f*_oc_) of the CTMAB-bentonite was determined using a Shimadzu TOC-V CPH organic-carbon analyser (Shimadzu Scientific Instruments, Kyoto, Japan). The X-ray diffraction (XRD) patterns were recorded using a Rigaku D/max-2550PC diffractometer with Cu Kα radiation, and the operations were carried out at a relative humidity of 60–70% and a temperature of 25 °C with a scanning rate of 4° (2*θ*) min^−1^. Samples were scanned from 1° to 15° (2*θ*), and the crystallographic spacing (*d*) was calculated using Bragg’s law, which can be described as *λ* = 2*d* sin*θ*. The FTIR spectra of organobentonites were recorded in the wavenumber range from 400 to 4000 cm^−1^ using a Nicolet 6700 FTIR Spectrometer (Thermo Fisher Scientific Inc., USA). The data analysis was carried out using IBM SPSS Statistics 20 (International Business Machines Corporation, USA) and OriginPro 8.5 (OriginLab Corporation, USA). The treatment differences were tested using the one-way ANOVA method (*α* = 0.05).

## Electronic supplementary material

Self-cleaning liner for halogenated hydrocarbon control in landfill leachate
