# Supplementary material for: Self-cleaning liner for halogenated hydrocarbon control in landfill leachate
Source: Sci Rep. 2017 Oct 26;7:14140. doi: 10.1038/s41598-017-14562-y (PMC5658343; doi:10.1038/s41598-017-14562-y)
Supplement: Supplementary file 1 — Self-cleaning liner for halogenated hydrocarbon control in landfill leachate [file 41598_2017_14562_MOESM1_ESM.pdf]

Supplementary Information

Self-cleaning liner for halogenated hydrocarbon control in landfill leachate

Shichong He[a, b], Lizhong Zhu[a, b*]

S1 Materials and Methods

S1.1 Sorption of halogenated hydrocarbon to CTMAB-bentonite

Briefly, 0.5 g of organobentonite and a 20-mL solution with varied concentrations of halogenated hydrocarbon were added to a batch of 22 mL glass tubes and sealed with a Teflon cap. After 3 hours of shaking at 25 °C, 150 rpm, the tubes were centrifuged at 3000 rpm for 15 minutes. Next, 10 mL of the supernatants was concentrated by liquid-liquid extraction using 5 mL n-hexane. Then, the organic phases were dehydrated using anhydrous sodium sulphate and then filtered through a 0.22 μm membrane filter. Three control batches were set to evaluate the loss of chemicals via volatilization or adsorption by glass tube and Teflon cap.

The repeat sorption ability of CTMAB-bentonite and CTMAB-bentonite mixed with iron was investigated. After sorption section, all tubes were centrifuged at 3000 rpm for 15 minutes and then 16 mL of supernatants was removed. Halogenated hydrocarbons in the supernatants were analysed as described in Section 2.4. Next, another 16-mL solution containing an initial concentration of halogenated hydrocarbons was added to teach tube. After being sealed with a Teflon cap, the tubes were shaken again at 25°C, 150 rpm. Three circulations were processed.

S1.2 Models for delineating the experimental sorption isotherm

To estimate the maximum capacity (Q_m) of materials in sorption, the Langmuir model was employed to delineate the experimental sorption isotherm according to the following equation:

$$C_e/Q_e = 1/(K_L \cdot Q_m) + C_e/Q_m \quad \text{(S1)}$$

where K_L is a constant related to the free energy of sorption ($L \cdot kg^{-1}$), C_e is the equilibrium concentration of chemicals in aqueous phase after the adsorption process ($mg \cdot L^{-1}$), and Q_e is the amount of chemicals adsorbed per unit of organobentonite ($mg \cdot g^{-1}$). K_L and Q_m were derived from the slope and intercept of the linear plot of C_e/Q_e against C_e, and Q_e was calculated as

$$Q_e = (C_0 - C_e) \cdot V/m \quad \text{(S2)}$$

where C_0 is the initial concentration of chloroform ($mg \cdot L^{-1}$), V is the solution volume (mL), and m is the sorbent mass (g).

A linear model was used to estimate the sorption capacity (K_d), and the sorption of halogenated hydrocarbons to CTMAB-bentonite could be calculated as

$$Q_e = K_d \cdot C_e \quad \text{(S3)}$$

where K_d is the sorption capacity ($L \cdot kg^{-1}$).

Additionally, Q could be calculated with the following equation:

$$Q_e = \frac{(C_0 - C_e) \cdot m}{V} \quad \text{(S4)}$$

As a result, the residual concentration of halogenated hydrocarbons could be calculated as

$$C_e = \frac{V}{V + K_d \cdot m} \cdot C_0 \quad \text{(S5)}$$

S1.3 Calculation method for concentration change of halogenated hydrocarbons

Q could be calculated using the following equation:

$$Q_e = \frac{(C_0 - C_e)\cdot m}{V} \quad \text{(S6)}$$

As a result, the residual concentration of halogenated hydrocarbons could be calculated as

$$C_e = \frac{V}{V + K_d \cdot m} \cdot C_0 \quad \text{(S7)}$$

The removal of halogenated hydrocarbons by different types of reagent could be described using the first-order kinetic model [1], which was written as:

$$C = C_0 \cdot e^{-kt} \quad \text{(S8)}$$

where *C* represents the concentration of the chemical at time *t*, e is the natural logarithm, *k* is the apparent reaction coefficient, and t is time.

The degradation of halogenated hydrocarbons is based on the consecutive reaction, and the change of the concentrations of halogenated hydrocarbons in the aqueous phase with time can be calculated as

$$C_{PCE} = C_{PCE,0} \cdot e^{-k_1 \cdot t} \quad \text{(S9)}$$

$$C_{TCE} = \left[\frac{C_{PCE,0} \cdot k_1}{k_2 - k_1} \cdot \left(e^{-k_1 \cdot t} - e^{-k_2 \cdot t}\right) + C_{TCE,0} \cdot e^{-k_2 \cdot t}\right] \quad \text{(S10)}$$

$$C_{DCE} = \left\{\frac{C_{PCE,0} \cdot \left[1 - \left(k_2 \cdot e^{-k_1 \cdot t} - k_1 \cdot e^{-k_2 \cdot t}\right)\right]}{k_2 - k_1} \cdot e^{-k_3 \cdot t} + C_{DCE,0} \cdot e^{-k_3 \cdot t}\right\} \quad \text{(S11)}$$

Combining Equation (S7) with Equations (S9) to (S11), the concentration change of halogenated hydrocarbons as removed by CTMAB-bentonite and iron mixture can be calculated as

$$C_{PCE} = C_{PCE,0} \cdot e^{-k_1 \cdot t} \cdot \frac{V}{V + K_{d,PCE} \cdot m} \quad \text{(S12)}$$

$$C_{TCE} = \left[\frac{C_{PCE,0}\cdot k_1}{k_2-k_1}\cdot\left(e^{-k_1\cdot t} - e^{-k_2\cdot t}\right) + C_{TCE,0}\cdot e^{-k_2\cdot t}\right]\cdot\frac{V}{V+K_{d,TCE}\cdot m} \quad \text{(S13)}$$

$$C_{DCE} = \left\{\frac{C_{PCE,0}\cdot\left[1-\left(k_2\cdot e^{-k_1\cdot t} - k_1\cdot e^{-k_2\cdot t}\right)\right]}{k_2-k_1}\cdot e^{-k_3\cdot t} + C_{DCE,0}\cdot e^{-k_3\cdot t}\right\}\cdot\frac{V}{V+K_{d,TCE}\cdot m} \quad \text{(S14)}$$

S1.4 Purchase of molecular interaction mechanisms in sorption

The molecular interaction mechanisms could be characterised through Linear Solvation Energy Relationship (LSER) approach [2,3]. For aqueous sorption, the equation could be write as:

$$\log K_d = c + rR_2 + s\pi_2^H + a\sum a_2^H + b\sum \beta_2^H + vV_x \quad \text{(S15)}$$

in which, R_2 presents the excess molecular refractivity of the sorbate that reflects the ability of the sorbate to interact with a sorbent through π or n-electron pairs; π_2^H presents the dipolarity/polarizing ability of the sorbate; $\sum a_2^H$ presents the hydrogen-bond acidity of the sorbent while $\sum \beta_2^H$ is the hydrogen-bond basicity of the sorbent; V_x is the McGowan's characteristic volume [4].

According to previous research[3], the equation S15 for organic chemicals sorbed to CTMAB-bentonite in the aqueous phase could be written as, which could be used to calculate the molecular interaction mechanisms in the sorption of certain chemical to CTMAB-bentonite:

$$\log K_d = -0.464 + 1.694R_2 - 0.435\pi_2^H + 0.591\sum a_2^H - 1.532\sum \beta_2^H + 2.277V_x \quad \text{(S16)}$$

References:

1 Wang, W. & Zhu, L. Effect of zinc on the transformation of haloacetic acids (HAAs) in drinking water. *J. hazard. mater.,* 2010 **174**, 40-46

2 Shih, Y.-h., Chou, S.-m., Peng, Y.-H. & Shih, M. Linear Solvation Energy Relationships Used To Evaluate Sorption Mechanisms of Volatile Organic Compounds with One

Organomontmorillonite under Different Humidities. *J. Chem. Eng. Data,* 2011, **56**, 4950-4955

3 Ruan, X. X., Zhu, L. Z., Chen, B. L., Qian, G. R. & Frost, R. L. Combined H-1 NMR and LSER study for the compound-specific interactions between organic contaminants and organobentonites. *J. Colloid Interface Sci.,* 2015, **460**, 119-12

4 Abraham, M. H. Scales of solute hydrogen-bonding: their construction and application to physicochemical and biochemical processes. *Chem. Soc. Rev.,* 1993, **22**, 73-83

Table S1 Total organic carbon (TOC) and inter layer spacing (*d*) of CTMAB-bentonite

Materials	TOC ($mg \cdot g^{-1}$)	*d* (nm)
bentonite	0.03	0.578
CTMAB-bentonite	206.7	1.465
CTMAB-bentonite after sorption	286.5	1.478
CTMAB-bentonite after degradation	204.6	1.465

Table S2 Molecular Descriptors of Organic Sorbates Used in This Study

Chemicals	R_2	π_2^{H}	a_2^{H}	β_2^{H}	V_x		rR_2	$s\pi_2^{H}$	$a\sum a_2^{H}$	$b\sum\beta_2^{H}$	vV_x
DCA	0.416*	0.64*	0.1*	0.11*	63.52***		0.807	-0.278	0.059	-0.168	144.6
TCA	0.499*	0.68*	0.13*	0.08*	75.76***		0.968	-0.296	0.077	-0.122	172.5
DCE	0.425*	0.41*	0.09*	0.005*	59.22***		0.824	-0.178	0.053	-0.008	134.8
TCE	0.524**	0.53**	0.12**	0.03**	71.46***		1.016	-0.230	0.071	-0.046	162.7

*: data obtained from Abraham, M. H., *Scales of solute hydrogen-bonding: their construction and application to physicochemical and biochemical processes*, Chem. Soc. Rev., 1993, **22**, 73-83.

: data obtained from Shih et al., *Linear Solvation Energy Relationships Used To Evaluate Sorption Mechanisms of Volatile Organic Compounds with One Organomontmorillonite under Different Humidities*, J. Chem. Eng. Data, 2011, **56(12), 4950-4955

***: data was calculated according to the methods described in Abraham, M. H. and J. C. McGowan, *The Use of characteristic Volumes to Measure Cavity Terms in Reversed Phase Liquid-chromatography*, Chromatographia, 1987, **23**(4), 243-246 and Abraham, M. H., *Scales of solute hydrogen-bonding: their construction and application to physicochemical and biochemical processes*, Chem. Soc. Rev., 1993, **22**, 73-83.

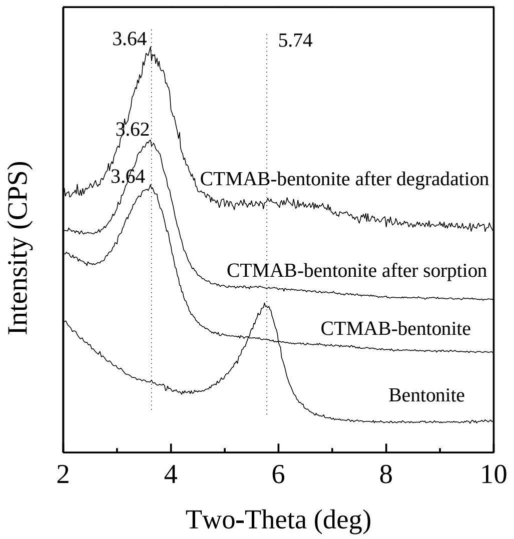

Figure S1 XRD patterns for bentonite and CTMAB-bentonites

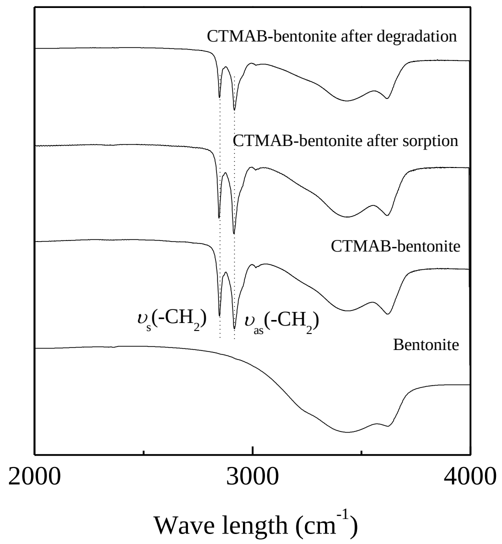

Figure S2 FTIR patterns for bentonite and CTMAB-bentonites

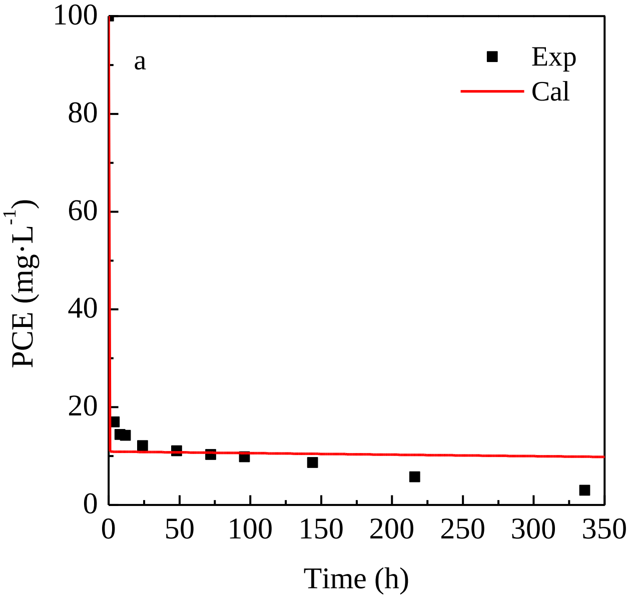

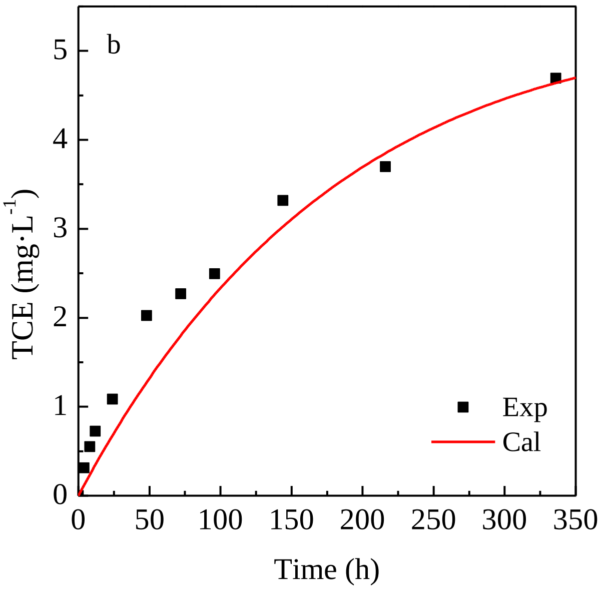

Figure S3 Experimental and calculated data for PCE degradation and TCE formation
a: degradation of PCE; b: formation of TCE

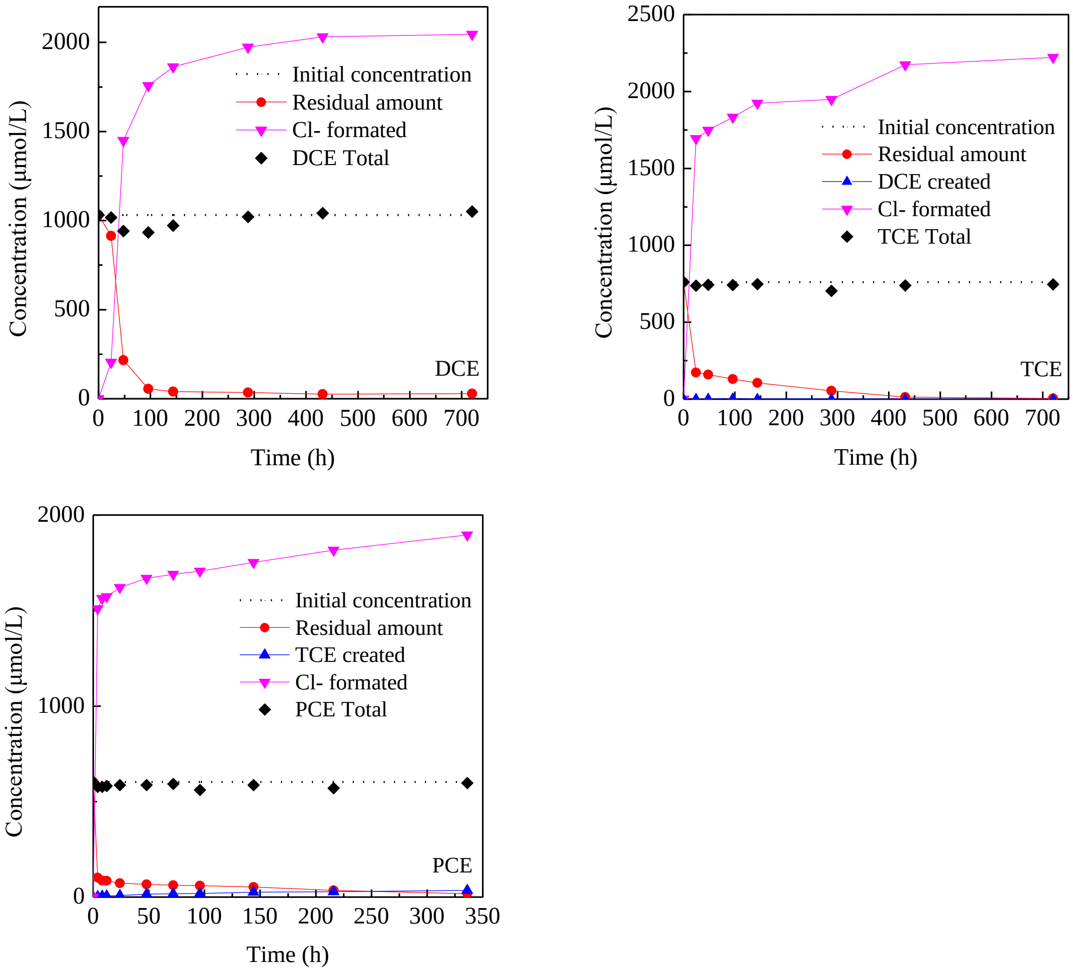

Figure S4 Degradation of DCE, TCE, PCE and the formation of the intermediate products

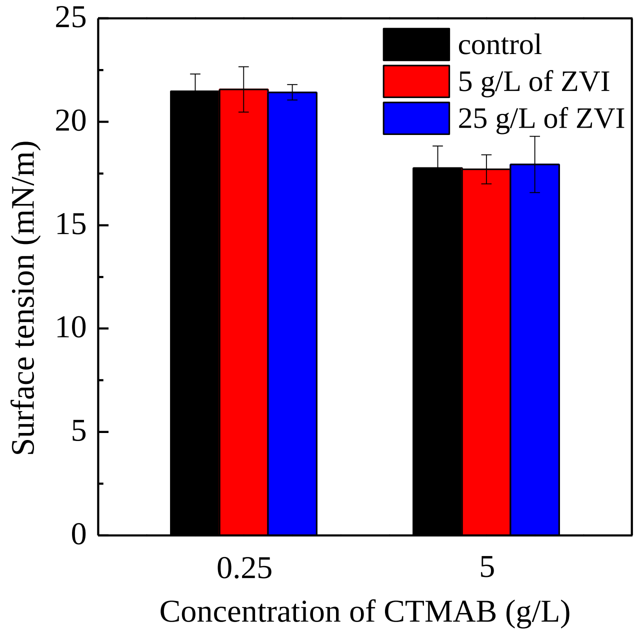

Figure S5 Surface tension of CTMAB solutions reacted with different mass of ZVI
